# Antenatal depression, obstetric outcomes, and post-partum depression: results from a longitudinal, real-world study

**DOI:** 10.1192/j.eurpsy.2023.1066

**Published:** 2023-07-19

**Authors:** M. Di Vincenzo, M. Ferrandino, V. Sollo, L. Landolfi, E. Mancuso, A. Di Cerbo, G. Sampogna, M. Luciano, A. Fiorillo

**Affiliations:** Department of Psychiatry, University of Campania “Luigi Vanvitelli”, Naples, Italy

## Abstract

**Introduction:**

Changes in physiological and hormonal balance occurring during pregnancy and post-partum period can have relevant implications on woman’s mental health. Up to 65% of pregnant women experience depressed mood, low self-esteem, cognitive impairment, fatigue, loss of appetite as well as suicidal ideation. Anxiety and depressive symptoms have been described as impactful on the newborn’s health at the time of delivery. Despite this, few evidence exist on this topic.

**Objectives:**

The present paper aimed at assessing: 1) prevalence and risk factors of antenatal depression (AD); 2) the impact of AD on adverse obstetric outcomes and the onset of post-partum depression.

**Methods:**

Pregnant outpatients attending the Department of Gynecology and Obstetrics of University of Campania “Luigi Vanvitelli” in Naples were asked to complete the Italian version of Edinburgh Postnatal Depression Scale (EPDS), a 10-item self-reported questionnaire developed as screening tool of postnatal depression up to one year after delivery. Sociodemographic, clinical and gestational information was collected at baseline.

**Results:**

A total of 268 pregnant women were recruited: 9.7% of them already suffered from depressive disorders and 22% from anxiety. EPDS mean total score was ≥10 in 36.2% of cases (97 out of 268). The presence of AD was longitudinally associated to a lower gestational age at the time of delivery and a higher 1 and 5 minutes APGAR scores. Moreover, AD was associated to a higher incidence of labor induction and the need of intensive care for the new-born. Finally, in our sample AD constituted a stable risk factor for EPDS scores within three days, one month and six months after delivery.

**Conclusions:**

The presence of depressive symptoms during pregnancy should deserve a higher clinical attention by health professionals, given the correlations with adverse obstetric outcomes and *post-partum* mental health. Training programmes should be encouraged and digital psychiatry could represent a strategy to monitor pregnant women at risk.

**Disclosure of Interest:**

None Declared

